# Psychosis as the Initial and Main Presentation of Graves’ Disease: A Case Report from Saudi Arabia

**DOI:** 10.7759/cureus.100181

**Published:** 2025-12-27

**Authors:** Abdulkarim O Alanazi, Enas M Aljohani, Reema T Alkalefah, Faisal H Alhadeedi, Haifa I Alkhuzayem

**Affiliations:** 1 Psychiatry, Prince Sultan Military Medical City, Riyadh, SAU; 2 Psychiatry, King Saud Bin Abdulaziz University for Health Sciences College of Medicine, Riyadh, SAU

**Keywords:** first-episode psychosis, graves’ disease, hyperthyroidism, psychoneuroendocrinology, secondary psychosis, thyrotoxicosis

## Abstract

Although psychosis is an uncommon manifestation of thyrotoxicosis, it may precede typical somatic features, leading to diagnostic delay. Recognition of its endocrine origin is crucial, as symptoms are usually reversible with appropriate treatment. A 29-year-old woman with no prior psychiatric or medical history presented with persecutory delusions, religious preoccupation, and auditory hallucinations in the absence of mood, cognitive, or classic somatic thyrotoxic symptoms. Examination revealed mild exophthalmos without other thyrotoxic signs. Laboratory tests showed suppressed thyroid-stimulating hormone (TSH; <0.005 mIU/L), elevated free triiodothyronine (T3; 30.5 pmol/L), and positive thyroid autoantibodies, confirming Graves’ disease. She was treated with carbimazole and atenolol, alongside low-dose olanzapine (5 mg daily). Her psychotic symptoms resolved completely concurrently with the normalization of thyroid function. This case illustrates that psychosis may be the initial and sole presentation of Graves’ disease. The close temporal relationship between biochemical improvement and symptom resolution supports a direct link between thyrotoxicosis and psychosis. Short-term, low-dose antipsychotics can be useful adjuncts during acute management but are rarely needed long-term once euthyroidism is achieved. Endocrine screening should be routinely performed in new-onset psychosis to identify reversible causes such as thyrotoxicosis. Early recognition and treatment of underlying hyperthyroidism ensures rapid psychiatric recovery and prevents unnecessary prolonged antipsychotic use.

## Introduction

Thyrotoxicosis is defined as the clinical state resulting from excessive circulating thyroid hormones, most often caused by Graves’ disease, but also toxic multinodular goiter, thyroiditis, or toxic adenoma [1]. It affects approximately 1.2% of the population, with overt hyperthyroidism more common in women aged 20 to 50 years [2]. The systemic manifestations are broad, typically involving cardiovascular abnormalities such as tachycardia and atrial fibrillation, metabolic and gastrointestinal disturbances including weight loss and diarrhea, and neuromuscular findings such as tremor and proximal myopathy [1,3]. Neuropsychiatric manifestations are also recognized; anxiety, irritability, emotional lability, restlessness, and insomnia are common, while more severe conditions such as mania, delirium, or frank psychosis occur rarely [4,5].

Historically, psychiatric disturbances have been more strongly associated with hypothyroidism, which has contributed to psychosis in hyperthyroidism being under-recognized and often overlooked in clinical practice [6]. Nevertheless, several case reports and reviews document psychosis as either a presenting or complicating feature of thyrotoxicosis, highlighting diagnostic and therapeutic challenges [7-9]. A recent review of 37 published cases found agitation, paranoia, and hallucinations to be the most frequently reported psychiatric symptoms, although psychosis remains distinctly uncommon compared to the more typical somatic features of thyrotoxicosis [1]. Regionally, reports from Saudi Arabia consistently emphasize the somatic and autonomic manifestations of thyrotoxicosis, such as neck swelling, palpitations, and tremors. However, no presentation of psychosis has been, to our knowledge, reported in the local literature. This emphasizes a knowledge gap in the recognition of such cases [10,11].

This case report aims to highlight an unusual presentation of Graves’ disease manifesting with isolated psychosis in the absence of classical thyrotoxic features. It underscores the importance of endocrine disorder screening in new-onset psychosis and demonstrates that timely recognition and treatment of hyperthyroidism enables full psychiatric recovery.

## Case presentation

A 29-year-old woman with no prior psychiatric or medical history and no family history of mental illness was brought to the emergency department by her family due to recent behavioral and perceptual changes. She had been functioning well before this episode, with no substance use or psychosocial stressors.

During the week preceding her emergency department visit, the patient’s condition markedly deteriorated. She developed persecutory delusions toward her brother, along with prominent religious preoccupations and delusional guilt. She also reported auditory hallucinations consisting of unfamiliar voices making derogatory and religiously themed comments. Her presentation included episodes of disorganized behavior, without evidence of manic, depressive, or cognitive symptoms.

The history revealed a three-month prodrome of gradual decline in social interaction and personal hygiene, which was punctuated by inappropriate smiling and staring episodes in the absence of agitation or disorganization. These changes fluctuated in intensity and were not associated with mood symptoms or external stressors.

On mental state examination, the patient appeared thin and anxious, with a disheveled appearance and poor hygiene. She was cooperative, maintained fair eye contact, and was fully oriented to time, place, and person. Her speech was coherent but occasionally tangential. Affect was restricted, and mood was anxious. Thought content revealed persecutory and guilt-related delusions with overvalued religious ideas. There was no thought blocking, loosening of associations, or formal thought disorder. Insight and judgment were impaired.

**Figure 1:**
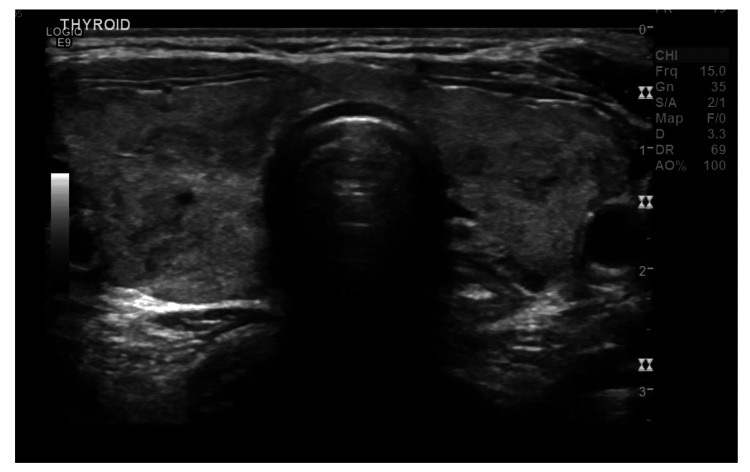
Thyroid ultrasonography demonstrated an enlarged heterogeneous gland and bilateral low-risk nodules without suspicious cervical lymphadenopathy.

**Figure 2:**
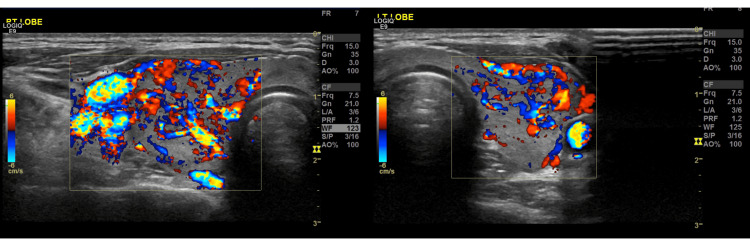
Thyroid ultrasonography showed bilateral increased vascularity.

Initial laboratory testing performed at a primary health clinic five days before psychiatric evaluation showed a suppressed thyroid-stimulating hormone (TSH) < 0.005 mIU/L, with elevated free triiodothyronine (T3) = 30.5 pmol/L and positive anti-thyroglobulin (20 IU/mL) and antithyroid peroxidase antibodies (141 IU/mL), consistent with autoimmune hyperthyroidism due to Graves’ disease (Table 1). Endocrinological examination identified mild exophthalmos and a diffuse, non-tender goiter; notably, there were no other classical systemic manifestations of thyrotoxicosis such as tremor, tachycardia, heat intolerance, weight loss, or palpitations. Thyroid ultrasonography (Figures 1, 2) demonstrated an enlarged heterogeneous gland with increased vascularity and bilateral low-risk nodules (Thyroid Imaging Reporting and Data System, Categories 2 and 3 (TI-RADS III and II)) without suspicious cervical lymphadenopathy.

**Table 1:** Patient's thyroid function test results Reference ranges: TSH = 0.4-4.0 mIU/L; free T4 = 9-23 pmol/L; free T3 = 3.1-6.8 pmol/L; antibody titers < 35 IU/mL considered negative. TSH: thyroid-stimulating hormone; T3: triiodothyronine; T4: thyroxine

Time point	TSH (mIU/L)	Free T4 (pmol/L)	Free T3 (pmol/L)	Anti-thyroglobulin (IU/mL)	Anti-thyroid peroxidase (IU/mL)
Upon presentation	< 0.005	N/A	30.5	20	141
Two days after starting antithyroid medication	< 0.005	9.2	37.8 – 39.4	N/A	N/A
45 days after the initial presentation	< 0.007	N/A	8.1	24	112

Further neurological work-up, including magnetic resonance imaging of the brain, electroencephalography, and cerebrospinal fluid analysis, was unremarkable, effectively excluding structural, infectious, or autoimmune encephalitic etiologies. During her admission, there were no fluctuations in consciousness or attention, ruling out delirium as a contributing factor.

The patient was managed collaboratively by psychiatry and endocrinology services. She was started on carbimazole and atenolol for thyrotoxicosis and commenced on low-dose olanzapine (5 mg daily) to address psychotic symptoms. Olanzapine was selected for its lower risk of extrapyramidal side effects and minimal pharmacologic interaction with antithyroid therapy.

Two days after initiating antithyroid treatment, repeat testing showed persistently suppressed TSH (<0.005 mIU/L), free thyroxine (T4) of 9.2 pmol/L, and free T3 between 37.8 and 39.4 pmol/L, indicating an early biochemical response (Table 1). Over the following weeks, her psychotic symptoms subsided in parallel with improvement in thyroid hormone levels. At 45 days after the initial presentation, her thyroid profile demonstrated TSH < 0.007 mIU/L, free T3 of 8.1 pmol/L, anti-thyroglobulin 24 IU/mL, and anti-thyroid peroxidase 112 IU/mL, consistent with near-euthyroid status (Table 1). Clinically, she showed complete remission of psychotic symptoms, restored insight, and full recovery of daily functioning. Olanzapine was gradually tapered and discontinued following sustained clinical stability. At the three-month follow-up, her mental state and thyroid function remained stable, with no recurrence of psychotic features. 

The patient’s biochemical trend demonstrated a marked reduction in free T3 levels from 30.5 pmol/L at presentation to 8.1 pmol/L at 45 days, while free T4 remained within the low-normal range and TSH persisted below the reference limit. This pattern reflects the expected physiological lag in pituitary recovery following treatment of thyrotoxicosis, where normalization of T3 precedes that of TSH. The close temporal association between T3 improvement and complete remission of psychotic symptoms supports a causal relationship between thyrotoxic hypermetabolism and the observed neuropsychiatric manifestations. Taken together, the clinical, laboratory, and temporal findings were most consistent with a secondary psychotic disorder directly related to Graves’ disease rather than a primary psychotic or affective illness. The final diagnosis was a psychotic disorder due to another medical condition, namely, Graves’ disease/thyrotoxicosis.

## Discussion

Thyroid hormones play a critical role in brain development and the regulation of neural activity throughout life. They influence synaptic transmission, neurotransmitter sensitivity, and cerebral metabolism, particularly within limbic regions involved in emotion and cognition [12,13]. Hyperthyroidism has been shown to alter beta (β)-adrenergic receptor density and enhance catecholaminergic sensitivity, leading to heightened arousal, irritability, and, in rare instances, psychosis [1]. These neurochemical changes explain why disturbances in thyroid hormone balance can precipitate a range of neuropsychiatric manifestations, most of which are reversible upon achieving euthyroid status [12].

Neuropsychiatric manifestations of thyrotoxicosis are well documented, though severe presentations such as psychosis remain distinctly uncommon, occurring in approximately 1% of cases [1,14]. The most frequently described symptoms include anxiety, insomnia, emotional lability, and irritability, while frank psychosis, manifesting as paranoia, religious delusions, or auditory hallucinations, occurs far less frequently [2,14,15]. In most reported cases, psychiatric manifestations were accompanied by overt systemic features of thyrotoxicosis, such as tremor, tachycardia, weight loss, or heat intolerance [4,5,8,9]. In contrast, the current case is unique in that psychotic symptoms were the sole presenting manifestation, occurring in the absence of classical somatic findings. This highlights the variable neuropsychiatric expression of Graves’ disease and the importance of considering thyroid dysfunction even when systemic features are minimal.

In a retrospective review, Brownlie et al. described 18 patients with acute psychosis associated with thyrotoxicosis, most of whom exhibited both somatic and psychiatric features [15]. Psychotic themes included paranoia, grandiosity, and religious preoccupation. The majority received short-term antipsychotic therapy, typically haloperidol or chlorpromazine in low to moderate doses (2-10 mg/day haloperidol equivalent), for periods ranging from one to three weeks, until euthyroidism was achieved. Similarly, Snabboon et al. and Saha et al. reported patients treated with low-dose risperidone (1-2 mg/day) or olanzapine (5-10 mg/day) for several weeks alongside methimazole and β-blockers, with full remission once thyroid hormone levels normalized [4,5]. In Rangappa et al., psychotic agitation resolved within three days of methimazole initiation, without continued antipsychotic therapy [2]. By contrast, Kothari et al. documented a patient whose psychosis persisted transiently after thyroidectomy and required short-term risperidone 2 mg/day for resolution [7]. Across reports, the consistent finding is that antipsychotics were used only briefly and at low doses, serving as adjuncts to endocrine treatment until hormonal equilibrium was restored.

In our patient, low-dose olanzapine (5 mg daily) was introduced concurrently with carbimazole and atenolol, leading to progressive improvement and complete remission within 45 days. The close correlation between biochemical normalization and psychiatric recovery supports the interpretation that psychosis was secondary to thyrotoxicosis rather than a primary psychiatric disorder. This aligns with prior literature indicating that once euthyroid, most patients can discontinue antipsychotics without relapse [1,2,5,15,16].

The principal diagnostic challenge lies in distinguishing secondary thyrotoxic psychosis from a primary psychiatric disorder [2]. Clues suggesting a secondary etiology include abrupt onset, fluctuating course, and concurrent autonomic or endocrine signs. Routine thyroid function testing in new-onset or atypical psychosis is therefore essential to prevent diagnostic delay and unnecessary long-term exposure to antipsychotics [16].

Treatment of thyrotoxic psychosis should focus primarily on correcting the underlying endocrine disorder through antithyroid agents and β-blockers, supplemented by short-term psychiatric management when needed [3,7]. The close temporal association between euthyroid restoration and symptom remission in this case mirrors findings across previous reports [1,2,5,15]. A multidisciplinary approach involving psychiatry, endocrinology, and neurology remains crucial for optimizing recovery.

Ultimately, this case underscores that psychosis can represent a reversible manifestation of hyperthyroidism, even in the absence of classical thyrotoxic signs. It reinforces the need for clinicians to maintain a high index of suspicion for endocrine causes in atypical or acute psychotic presentations. Routine thyroid screening facilitates timely diagnosis, prevents unnecessary pharmacologic exposure, and improves outcomes. To our knowledge, this is the first reported case from Saudi Arabia of Graves’ disease presenting primarily with psychosis, emphasizing the need for greater awareness among regional clinicians [10,11].

## Conclusions

As this case shows, psychosis secondary to thyrotoxicosis is a rare but clinically significant reversible manifestation of Graves’ disease that can occur even in the absence of overt systemic signs. This case emphasizes the importance of maintaining diagnostic vigilance for endocrine etiologies in acute or atypical psychotic presentations, as timely treatment of the underlying thyroid dysfunction can result in complete remission. While short-term antipsychotic therapy may provide symptomatic relief, definitive improvement is achieved through restoration of euthyroid status. Further research exploring the neurobiological mechanisms linking thyroid hormone dysregulation to psychosis and establishing evidence-based guidelines for the use and duration of adjunctive antipsychotics in this context.
